# Potential Bio-Control Agent from *Rhodomyrtus tomentosa* against *Listeria monocytogenes*

**DOI:** 10.3390/nu7095346

**Published:** 2015-09-07

**Authors:** Grace Fiyinfoluwa Odedina, Kitiya Vongkamjan, Supayang Piyawan Voravuthikunchai

**Affiliations:** 1 Department of Microbiology, Faculty of Science, Prince of Songkla University, Hat Yai, Songkhla 90112, Thailand; E-Mail: odedinagrace@gmail.com; 2 Excellent Research Laboratory on Natural Products, Faculty of Science and Natural Product Research Center of Excellence, Prince of Songkla University, Hat Yai, Songkhla 90112, Thailand; 3 Department of Food Technology, Faculty of Agro-Industry, Prince of Songkla University, Hat Yai, Songkhla 90112, Thailand; E-Mail: kitiya.v@psu.ac.th

**Keywords:** *Listeria monocytogenes*, *Rhodomyrtus tomentosa*, antibacterial activity, salt tolerance, foodborne, bio-control

## Abstract

*Listeria monocytogenes* is an important foodborne pathogen implicated in many outbreaks of listeriosis. This study aimed at screening for the potential use of *Rhodomyrtus tomentosa* ethanolic leaf extract as a bio-control agent against *L. monocytogenes*. Twenty-two *L. monocytogenes* isolates were checked with 16 commercial antibiotics and isolates displayed resistance to 10 antibiotics. All the tested isolates were sensitive to the extract with inhibition zones ranging from 14 to 16 mm. Minimum inhibitory concentration (MIC) and minimum bactericidal concentration (MBC) values ranged from 16 to 32 µg/mL and 128 to 512 µg/mL, respectively. Time-kill assay showed that the extract had remarkable bactericidal effects on *L. monocytogenes*. The extract at a concentration of 16 µg/mL reduced tolerance to 10% NaCl in *L. monocytogenes* in 4 h. Stationary phase *L. monocytogenes* cells were rapidly inactivated by greater than 3-log units within 30 min of contact time with *R. tomentosa* extract at 128 µg/mL. Electron microscopy revealed fragmentary bacteria with changes in the physical and morphological properties. Our study demonstrates the potential of the extract for further development into a bio-control agent in food to prevent the incidence of *L. monocytogenes* contamination.

## 1. Introduction

*Listeria monocytogenes* is a Gram-positive, rod-shaped bacterium implicated in a severe, opportunistic foodborne infection, listeriosis [1]. The pathogen is ubiquitous with isolations from humans and animals [2] as well as from raw and ready-to-eat foods [3]. *L. monocytogenes* remains a nuisance to the food industry because of its persistence on food production surfaces and its contamination of food products. Policies varying in different countries are put in place to control the number of *L. monocytogenes* cells in ready-to-eat food. In the United States, there is a “zero tolerance policy” for the presence of *L. monocytogenes* cells in 25 g of ready-to-eat food [4,5]. Meanwhile, the recent increase in food changing habits and technological advancements for the longer shelf life of food products have contributed to the growth, survival, and persistence of the pathogen in food at extreme conditions such as high salinity. Consumer awareness to health hazards associated with synthetic antimicrobials has revolutionized the research focus on food antimicrobials from biological origins to effectively control *L. monocytogenes*, as they are generally recognized as safe.

Herbal plants and their components have been consumed as food for decades and have gained awareness for their therapeutic potencies, as well as their growth and health benefits [6]. Numerous plant antimicrobials with anti-*Listeria* activity have been previously reported, including peel extract of *Punica granatum* (pomegranate) [7], seed extract of *Garcinia kola* (bitter kola) [8], calyx extract of *Hibiscus sabdariffa* (roselle) [9], and leaf extract of *Syzygium aromaticum* (clove) [10]. As reviewed by Tajkarimi *et al.* [11], many plant antimicrobials present with setbacks including high cost, strong colour, and odour because of their usually high effective dose. Research is still ongoing to source for plant antimicrobials which may be effective against *L. monocytogenes* at lower extract concentrations with a desired level of acceptability.

*Rhodomyrtus tomentosa* is a flowering plant in the Myrtaceae family, native to Southeast Asia. Extracts from the leaves have been previously reported to display interesting antibacterial activity against many Gram-positive pathogens [12,13,14,15]. The ethanol extract of *R. tomentosa* leaves displayed inhibitory activity against *L. monocytogenes* in a preliminary study [12] and remarkable activity against foodborne pathogens including *Bacillus cereus* [14] and *Staphylococcus aureus* [15]. The aim of the present study, therefore, was to further investigate the antibacterial activity of *R. tomentosa* ethanolic leaf extract against *L. monocytogenes*, which could be developed into a bio-control strategy in food to prevent *L. monocytogenes* contamination.

## 2. Experimental Section

### 2.1. Bacterial Strains and Culture Condition

Nineteen *L. monocytogenes* isolates, including nine isolates from ready-to-eat food and 10 isolates from food processing plant environments were used in this study [16,17]. Reference strains consisting of *L. monocytogenes* FSL R2-574 (food) and FSL F2-501 (human) were obtained from the Food Safety Lab, Cornell University, Ithaca, New York, and *L. monocytogenes* Scott A (human) was kindly provided by Prof. Dr. Catherine Nettles Cutter, Department of Food Science, Pennsylvania State University. All the bacterial cultures were stored in Tryptic Soy Broth (TSB; Difco, Le Port de claix, France) supplemented with 30% glycerol and kept at −80 °C. Each bacterial culture was grown on Tryptic Soy Agar (TSA; Difco Le Port de claix, France) at 37 °C for 24 h prior to use.

### 2.2. Plant Extract Preparation

The classified reference voucher specimen of *Rhodomyrtus tomentosa* leaves (NPRC0057) deposited in the Faculty of Traditional Thai Medicine, Prince of Songkla University, Thailand, was extracted according to the previously published methodology [12]. Briefly, *R. tomentosa* leaves were dried, grounded, extracted with 95% ethanol, and subsequently evaporated of the ethanol using a rotary evaporator. The extract was dissolved in 100% dimethyl sulfoxide (DMSO; Bangkok, Thailand) to obtain a concentration of 100 g/L and kept at −20 °C until use.

### 2.3. Antibiotic Susceptibility Screening of Isolates

Antibiotic susceptibility patterns of *L. monocytogenes* were determined using 16 commercial antibiotics commonly used in human and veterinary medicine, following a standard agar disc susceptibility assay proposed by the Clinical and Laboratory Standards Institute (CLSI) [18] with a slight modification. Three to five bacterial colonies from an 18 h culture were transferred into TSB and incubated at 37 °C for 4 h. The bacterial suspension was adjusted to McFarland standard turbidity of No. 0.5 and aliquot was spread plated on the surface of a well-solidified Mueller-Hinton agar (MHA; Difco Le Port de claix, France) plate, using a sterile cotton swab. The inoculated plate was air dried in a biosafety cabinet for 10 min and subsequently seeded with antibiotic discs (Oxoid, Hampshire, England) consisting of penicillin G (10 units), ampicillin (10 µg), gentamycin (10 µg), sulfamethoxazole-trimethoprim (25 µg), erythromycin (15 µg), chloramphenicol (30 µg), amikacin (30 µg), streptomycin (10 µg), vancomycin (30 µg), ciprofloxacin (5 µg), norfloxacin (10 µg), teicoplanin (30 µg), fusidic acid (10 µg), kanamycin (30 µg), imipenem (10 µg), and ceftazidime (30 µg). The plate was incubated at 37 °C for 18 to 24 h and zones of inhibition were measured using a vernier caliper. The means of inhibition zones and standard errors from two independent studies were recorded and compared with the respective antibiotic interpretative standards for Staphylococci as outlined in CLSI, except for ampicillin and penicillin G, for which interpretative standards were specifically provided for *L. monocytogenes* [18].

### 2.4. Antibacterial Activity of Rhodomyrtus tomentosa Ethanolic Leaf Extract against Listeria monocytogenes Using Agar Disc Diffusion Assay

Preliminary screening of *L. monocytogenes* isolates with ethanolic leaf extract of *R. tomentosa* was set up using the agar disc diffusion assay [18] with a slight modification. Adjusted bacterial suspension was spread plated on MHA following the procedure described in Section 2.3 above. Paper discs containing 2.5 mg of the extract were prepared by pipetting 10 µL aliquot of a 250 mg/mL solution of the extract onto sterile 6 mm Whatman filter paper discs and dried at 37 °C overnight. Discs containing 1% DMSO were used as control. Discs were seeded onto the surface of MHA followed by incubation at 37 °C for 18 to 24 h. The experiment was set up in two independent studies and means of inhibition zones ± standard error were calculated.

### 2.5. Determination of Minimum Inhibitory Concentration (MIC) and Minimum Bactericidal Concentration (MBC) of Rhodomyrtus tomentosa Ethanolic Leaf Extract against Listeria monocytogenes

Antibacterial activity of *R. tomentosa* ethanolic leaf extract was determined against *L. monocytogenes* isolates following a slightly modified broth microdilution method outlined by CLSI [18]. A four-hour culture of *L. monocytogenes* was grown in TSB as described in Section 2.3 above and adjusted to McFarland standard turbidity of No. 0.5. The suspension was diluted 1:100 in 0.85% normal saline solution (NSS) to a final concentration of approximately 10^6^ CFU/mL. One hundred microliters of *R. tomentosa* ethanolic leaf extract, prepared in four folds of the highest concentration, were serially diluted to two folds of varying concentrations (512 µg/mL to 8 µg/mL) in a sterile 96-well flat bottom microtitre plate and subsequently inoculated with 100 µL of the bacterial cells in triplicates. The plant extract at various concentrations without the bacterial cells, 1% DMSO, and TSB media alone were included as controls followed by incubation at 37 °C for 24 h. The minimum inhibitory concentration (MIC) was recorded as the lowest concentration that produced a complete inhibition of visible growth in the microtitre plate. Ten microliter aliquots were spotted on TSA plates from wells displaying no visible growth and the plates were incubated at 37 °C for 24 h. The minimum bactericidal concentration (MBC) of the extract was recorded as the lowest concentration completely preventing bacterial growth on TSA plates after incubation at 37 °C for 24 h. Penicillin G (Sigma, St. Louis, MO, USA) was used as reference antibiotic. All experiments were set up in triplicates for two independent studies.

### 2.6. Time-Kill Assay

The rate of killing by *R. tomentosa* ethanolic leaf extract against *L. monocytogenes* actively dividing cells in nutrient medium was investigated using a time-kill assay [14,19]. Multidrug-resistant food isolates including *L. monocytogenes* PSU-KV-133LM, *L. monocytogenes* FSL R2-574, and *L. monocytogenes* Scott A were selected for the study. Bacterial inoculum (10^6^ CFU/mL) from growing culture in TSB prepared as described in Section 2.5 above was supplemented with the extract at ½ MIC, MIC, 2 MIC, and 4 MIC and subsequently incubated at 37 °C for 24 h. Surviving cells were enumerated at time 0, 2, 4, 8, 12, and 24 h using a serial dilution and plate count method. Control cultures with 0.1% DMSO were incubated under the same condition. The experiment was set up in duplicates for three independent studies.

### 2.7. Test for Salt Tolerance

The inability of *L. monocytogenes* cells treated with *R. tomentosa* ethanolic leaf extract in the presence of 10% sodium chloride (NaCl; Emsure, Billerica, MA, USA) to grow on TSA was tested following Carson *et al.*’s [20] method with slight modifications. *L. monocytogenes* representatives used in Section 2.6 above were used in the study. Bacterial culture (10^6^ CFU/mL) was prepared in TSB as described in Section 2.5 above. This was treated with ½ MIC, MIC, 2 MIC, and 4 MIC of the extract and 0.1% DMSO in two groups consisting of either presence or absence of 10% NaCl in 96-well microtitre plates. Plates were incubated at 37 °C in a shaker (Forma Scientific, Inc., Ohio, OH, USA) at 150 rpm. One hundred microliter aliquot was sampled at 0, 2, 4, 8, 12, and 24 h and surviving cells were enumerated on TSA plates by serial dilution and plate count method. The study was set up in duplicates for three independent studies.

### 2.8. Determination of Bacterial Inactivation Using Rhodomyrtus tomentosa Ethanolic Leaf Extract

Bacterial inactivation assay was set up to study the behavior of *L. monocytogenes* cells in the stationary phase of growth when treated with *R. tomentosa* ethanolic leaf extract in normal saline solution, following the protocol by Pattanayaiying *et al.* [21] with slight modifications. The stationary phase of *L. monocytogenes* was pre-determined from a bacterial growth curve monitored for 24 h. Cells attained stationary phase from about 8 to 24 h and mid-stationary phase cells, cultured for 16 h, was used in the study. *L. monocytogenes* representatives used in Section 2.6 above were grown in TSB supplemented with 0.6% yeast extract (LabM, Lancashire, UK) (TSB-YE) overnight for 16 h. Cells were harvested by centrifugation at 8000 rpm for 15 min, washed five times using sterile NSS, and adjusted to a final concentration of approximately 10^8^ CFU/mL in NSS. Cell suspensions were supplemented with the extract at ½ MIC, MIC, 2 MIC, and 4 MIC and the control with 0.1% DMSO in flat bottom 96-well microtitre plates, followed by incubation at 37 °C for 12 h. Samples were taken at time 0, 0.5, 1, 2, 4, and 12 h and the remaining bacterial population was determined by serial dilution and plate count method. All experiments were set up in duplicates for three independent studies.

### 2.9. Scanning Electron Microscopy (SEM)

The effects of *R. tomentosa* ethanolic leaf extract on *L. monocytogenes* Scott A cells were observed using electron microscopy. Cells were grown in TSB-YE medium for 4 h and adjusted to approximately 10^6^ CFU/mL. The culture was supplemented with the extract at MIC and 2 MIC. The control consisted of bacterial culture incorporated with 0.1% DMSO. The tubes were incubated at 37 °C for 12 h in a shaker at 150 rpm. After incubation, cells were harvested by centrifugation at 8000 rpm for 5 min, washed three times with NSS, subsequently coated onto slides and fixed with a 3% glutaraldehyde solution (Sigma-Aldrich, St. Louis, MO, USA) for 2 h. The slides were washed three times with NSS and dehydrated at room temperature with various ethanol concentrations consisting of 30%, 50%, 70%, 90%, and 100% for 15 min at each step. Cells were dried further and sputter-coated with gold palladium under vacuum and the morphology was observed and photographed with FEI Quanta 400 FEG Scanning Electron Microscope.

## 3. Results

### 3.1. Antibiotic Susceptibility Pattern of Listeria monocytogenes Isolates from Ready-to-Eat Food and Food Processing Plant Environments

All *L. monocytogenes* investigated displayed resistance to ceftazidime (Table 1). Resistance was 4.5% to norfloxacin, 5% to penicillin, ampicillin, amikacin, streptomycin, kanamycin, and gentamicin and 18% to ciprofloxacin and fusidic acid. Intermediate susceptibility was 4.5%, 5%, 5%, 18%, and 82% to norfloxacin, erythromycin, chloramphenicol, streptomycin, and fusidic acid, respectively. In addition, 27.3% of the isolates displayed resistance to at least two antibiotics. However, all the isolates were susceptible to sulfamethoxazole-trimethoprim, imipenem, teicoplanin, and vancomycin.

**Table 1:** Antibiotic susceptibility patterns of *Listeria monocytogenes* isolates from ready-to-eat food and food processing plant environments.

Antibiotics	Dose (µg/disc)	Sensitivity of the Isolates (%) *^a^*		
		R	I	S
Sulfamethoxazole-trimethoprim	25	0	0	100
Imipenem	10	0	0	100
Teicoplanin	30	0	0	100
Vancomycin	30	0	0	100
Penicillin G	10 Units	5	0	95
Ampicillin	10	5	0	95
Gentamicin	10	5	0	95
Erythromycin	15	0	5	95
Kanamycin	30	5	0	95
Amikacin	30	5	0	95
Chloramphenicol	30	0	5	95
Norfloxacin	10	4.5	4.5	91
Ciprofloxacin	5	18	0	82
Streptomycin	10	5	18	77
Fusidic acid	10	18	82	0
Ceftazidime	30	100	0	0

***^a^*** Interpretation of antibiotic susceptibility based on CLSI [18]; R: Resistance, I: Intermediate, S: Susceptible. Total of 22 *L. monocytogenes* isolates were tested.

### 3.2. Inhibitory Effects of Rhodomyrtus tomentosa Ethanolic Leaf Extract on Listeria monocytogenes Isolates from Ready-to-Eat Food and Food Processing Plant Environments

At a concentration of 2.5 mg/disc, *R. tomentosa* ethanolic leaf extract produced large inhibition zones ranging from 14 to 16 mm against all tested isolates with an average value of 15.2 ± 0.44 mm, 14.7 ± 0.35 mm, 13.5 ± 0.21 mm, and 18.6 ± 0.00 mm against 19 isolates, *L. monocytogenes* FSL R2-574, FSL F2-501, and Scott A, respectively (Table 2).

**Table 2:** Antibacterial activity of *Rhodomyrtus tomentosa* ethanolic leaf extract against *Listeria monocytogenes* isolates from ready-to-eat food and food processing plant environments.

Isolate Name	Sources (Serotype)	Inhibition Zone (mm) *^b^*	MIC (µg/mL)	MBC (µg/mL)	Reference(s)
PSU-KV-008LM	FPE (1/2b, 3b, 4b, 4d, 4e) *^a^*	14.60 ± 0.14	16	256	[16]
PSU-KV-009LM	FPE (1/2b, 3b, 4b, 4d, 4e) *^a^*	14.30 ± 0.42	16	128	[16]
PSU-KV-030LM	FPE (1/2b, 3b, 4b, 4d, 4e) *^a^*	15.05 ± 0.78	16	256	[16]
PSU-KV-031LM	FPE (1/2b, 3b, 4b, 4d, 4e) *^a^*	15.55 ± 0.21	16	256	[16]
PSU-KV-032LM	FPE (1/2b, 3b, 4b, 4d, 4e) *^a^*	15.35 ± 1.20	16	256	[16]
PSU-KV-033LM	FPE (1/2b, 3b, 4b, 4d, 4e) *^a^*	14.25 ± 0.49	16	256	[16]
PSU-KV-036LM	FPE (1/2b, 3b, 4b, 4d, 4e) *^a^*	15.70 ± 0.00	16	256	[16]
PSU-KV-038LM	FPE (1/2b, 3b, 4b, 4d, 4e) *^a^*	14.90 ± 1.13	16	256	[16]
PSU-KV-039LM	FPE (1/2b, 3b, 4b, 4d, 4e) *^a^*	15.60 ± 0.00	16	256	[16]
PSU-KV-137LM	Ready-to-eat food	15.75 ± 0.21	32	512	[17]
PSU-KV-105LM	Ready-to-eat food	15.55 ± 0.21	32	256	[17]
PSU-KV-108LM	Ready-to-eat food	14.95 ± 0.92	32	256	[17]
PSU-KV-111LM	Ready-to-eat food	15.05 ± 0.35	32	256	[17]
PSU-KV-116LM	Ready-to-eat food	15.15 ± 1.06	32	256	[17]
PSU-KV-120LM	Ready-to-eat food	15.60 ± 0.42	32	128	[17]
PSU-KV-122LM	Ready-to-eat food	15.10 ± 0.99	32	128	[17]
PSU-KV-127LM	Ready-to-eat food	15.30 ± 0.00	32	256	[17]
PSU-KV-133LM	Ready-to-eat food	15.00 ± 0.71	32	128	[17]
PSU-KV-148LM	Ready-to-eat food	15.10 ± 0.99	16	128	[17]
FSLR2-574	Food (4b)	14.65 ± 0.35	32	128	[22]
FSLF2-501	Human (4b)	13.35 ± 0.21	32	128	[22]
Scott A	Human (4b)	18.60 ± 0.00	32	128	[23]

*^a^* Serotypes classified by multiplex-PCR; (FPE) is food processing plant environments; ***^b^*** Values are the mean ± duplicate determinations from treatment with the extract at a concentration of 2.5 mg/disc.

### 3.3. Minimum Inhibitory Concentration (MIC) and Minimum Bactericidal Concentration (MBC) of Rhodomyrtus tomentosa Ethanolic Leaf Extract against Listeria monocytogenes Isolates from Ready-to-Eat Food and Food Processing Plant Environments

Minimum inhibitory concentrations and minimum bactericidal concentrations of the extract against isolates ranged from 16 to 32 µg/mL and 128 to 512 µg/mL, respectively (Table 3). MIC_50_ (MIC at which 50% of the isolates were inhibited) and MIC_90_ (MIC at which 90% of the isolates were inhibited) were 16 µg/mL and 32 µg/mL, respectively. MBC_50_ (MBC at which 50% of the isolates were killed) and MBC_90_ (MBC at which 90% of the isolates were killed) were both 256 µg/mL. In addition, the extract displayed similar MIC and MBC values of 32 µg/mL and 128 µg/mL, respectively, against the three reference strains investigated. The MIC value of penicillin G against isolates ranged from 0.125 to 1 µg/mL, while MIC_50_ and MIC_90_ were 0.250 µg/mL and 1 µg/mL, respectively. The MBC values of penicillin G against the isolates ranged from 1 to 4 µg/mL and MBC_50_ and MBC_90_ values were 2 µg/mL and 4 µg/mL, respectively. The MIC and MBC values of Penicillin G against *L. monocytogenes* Scott A were 2 µg/mL and >4 µg/mL, respectively, while these values against both FSL R2-574 and FSL F2-501 were 0.25 µg/mL and 1 µg/mL, respectively.

**Table 3:** Minimum inhibitory concentration (MIC) and minimum bactericidal concentration (MBC) of *Rhodomyrtus tomentosa* ethanolic leaf extract against *Listeria monocytogenes* isolates from ready-to-eat food and food processing plant environments.

	MICs and MBCs (µg/mL) *Listeria monocytogenes*											
	Isolates (*n* = 19)						FSL R2-574		FSL F2-501		Scott A	
Antibacterial agent	MIC	MIC_50_	MIC_90_	MBC	MBC_50_	MBC_90_	MIC	MBC	MIC	MBC	MIC	MBC
*Rhodomyrtus tomentosa* extract	16–32	16	32	128–512	256	256	32	128	32	128	32	128
Penicillin G	0.125–1	0.25	1	1–4	2	4	0.25	1	0.25	1	2	>4

### 3.4. Time-Kill Curve of Listeria monocytogenes Treated with Rhodomyrtus tomentosa Ethanolic Leaf Extract

**Figure 1:**
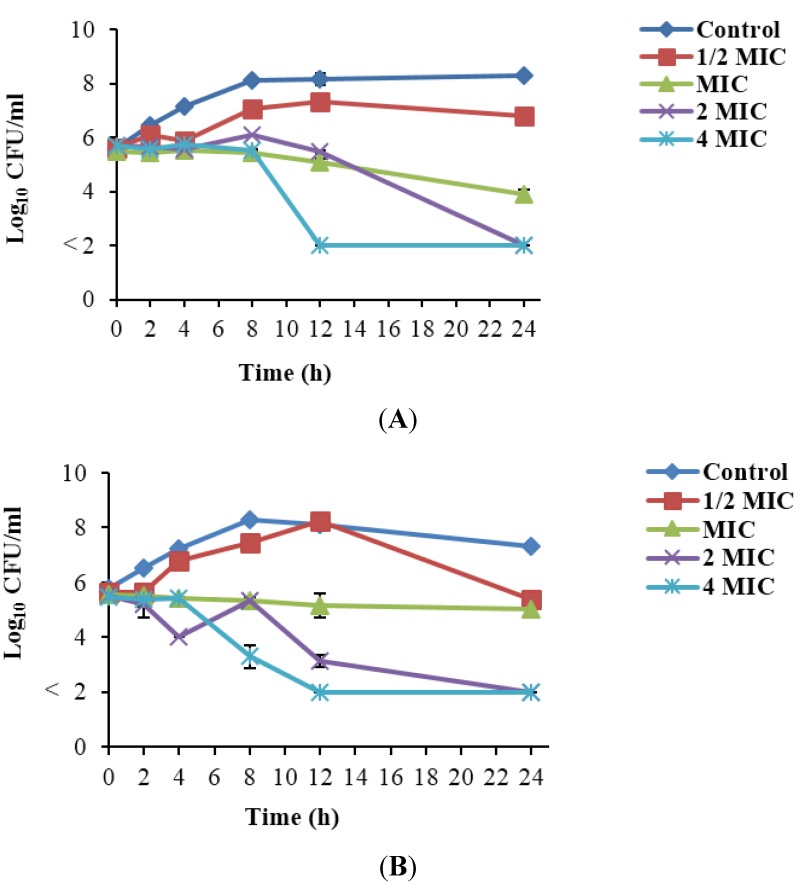

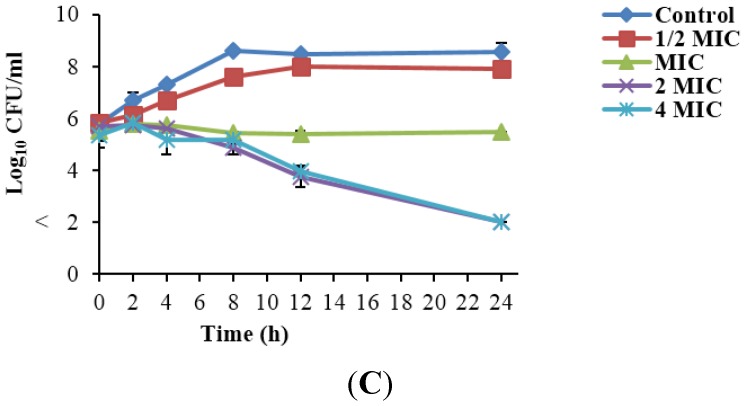
Time-kill curve of *Listeria monocytogenes*, Scott A (**A**); FSL R2-574 (**B**); and PSU-KV-133 (**C**), after treatment with *Rhodomyrtus tomentosa* ethanolic leaf extract at various concentrations, as well as the control with 0.1% dimethyl sulfoxide. The detection limit was 10^2^ CFU/mL.

A reference strain, *L. monocytogenes* Scott A, widely used for ascertaining the efficacy of food processing and preservation techniques, one food strain, FSL R2-574 and one food isolate, PSU-KV-133LM, showing resistance to two antibiotics, were selected for time-kill studies (Figure 1). At ½ MIC, *R. tomentosa* ethanolic leaf extract inhibited growth in *L. monocytogenes* Scott A for 4 h and in FSL R2-574 and PSU-KV-133LM for 2 h. At the MIC (32 µg/mL), more than a 1.5-log reduction in Scott A cells was observed within 24 h while cells of FSL R2-574 and PSU-KV-133LM were maintained in the lag phase for 24 h. At 2 MIC, the cells were reduced by more than 3-log units within 24 h. At the MBC (4 MIC), there was a reduction in cell numbers beyond the detection limit within 12 h in Scott A and FSL R2-574, and within 24 h in PSU-KV-133LM. There was no inhibition of growth in cells treated with 0.1% DMSO.

### 3.5. Reduction of Salt Tolerance in Listeria monocytogenes by Rhodomyrtus tomentosa Ethanolic Leaf Extract

**Figure 2:**
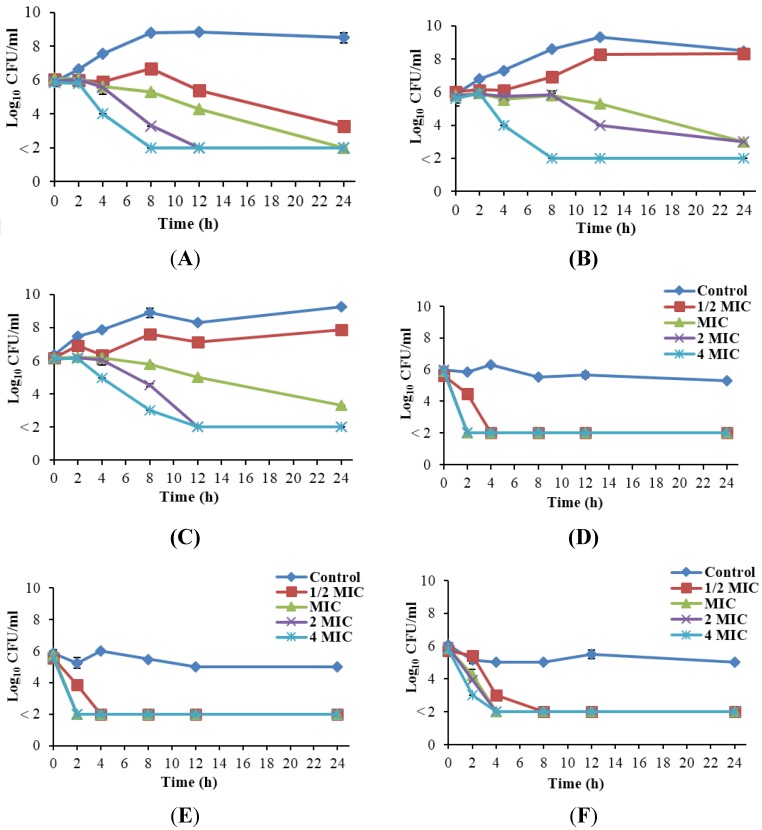
Numbers of *Listeria monocytogenes* cells, Scott A (**A**); FSL R2-574 (**B**); and PSU-KV-133 (**C**), able to form colonies on tryptic soy agar after treatment with *Rhodomyrtus tomentosa* ethanolic leaf extract at various concentrations, and Scott A (**D**); FSL R2-574 (**E**); and PSU-KV-133 (**F**) after treatment with the extract at various concentrations + 10% NaCl. The detection limit was 10^2^ CFU/mL.

*R. tomentosa* ethanolic leaf extract reduced salt tolerance in *L. monocytogenes* at ½ MIC (16 µg/mL) in 4 h (Figure 2). However, cells in the control group survived in the presence of 10% NaCl for 24 h in all tested isolates. Furthermore, cells were killed rapidly and more effectively with the plant extract in the presence of 10% NaCl than in the absence of salt.

### 3.6. Inactivation of Listeria monocytogenes Treated with Rhodomyrtus tomentosa Ethanolic Leaf Extract

**Figure 3:**
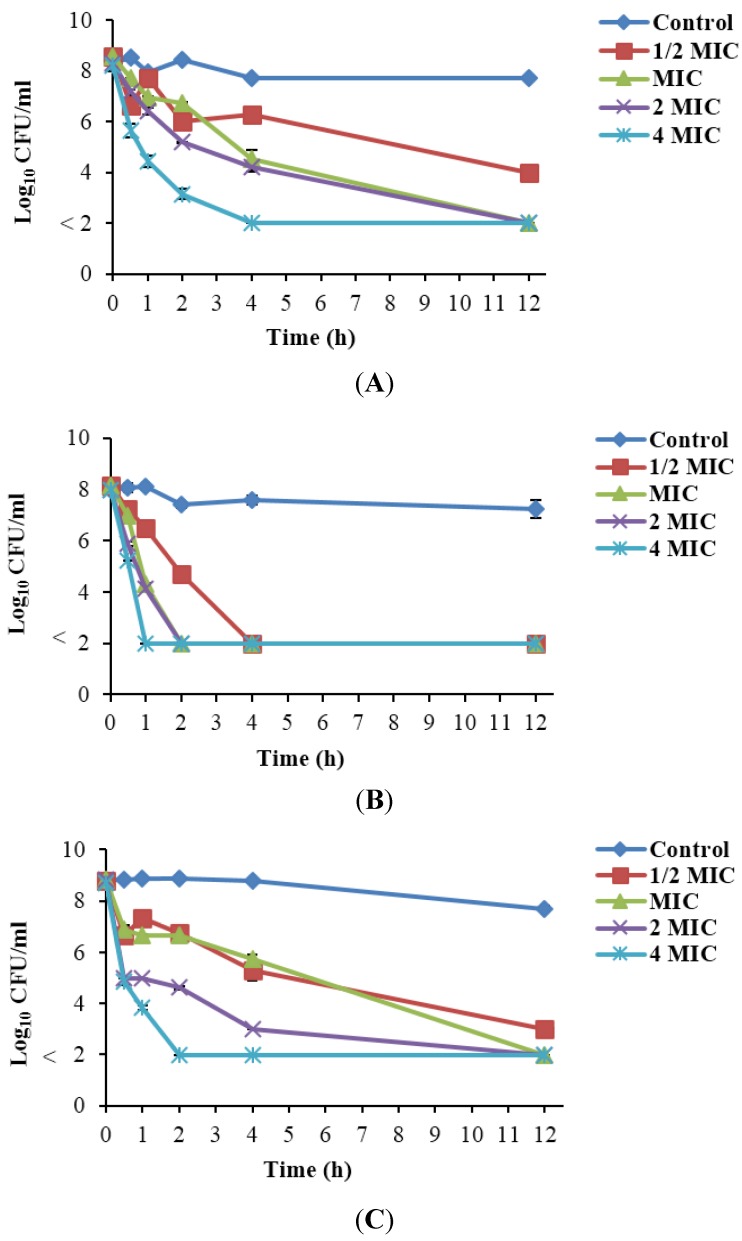
Inactivation of *Listeria monocytogenes* Scott A (**A**); FSL R2-574 (**B**); and PSU-KV-133 (**C**) using *Rhodomyrtus tomentosa* ethanolic leaf extract at various concentrations, as well as the control with 0.1% dimethyl sulfoxide. Cells in the stationary phase of growth were treated with the extract in sterile 0.85% normal saline solution and surviving cells were enumerated at time intervals. The detection limit was 10^2^ CFU/mL.

Stationary phase cells were treated with the plant extract in suspension at different concentrations. At ½ MIC, cells were inactivated by greater than 4-log units within 12 h of incubation in all tested isolates (Figure 3). At the MIC, a greater than 3-log reduction was observed within 4 h of treatment in *L. monocytogenes* Scott A and PSU-KV-133LM, while reduction was beyond detection limit within 2 h in FSL R2-574. Similarly, at 2 MIC, Scott A and PSU-KV-133LM were inactivated by greater than 3-log units in 2 h, while FSL R2-574 was inactivated beyond detection limit in 2 h. At the MBC, cells were inactivated beyond the detection limit (more than 6-log reduction) in 1 h, 2 h, and 4 h of incubation in FSL R2-574, PSU-KV-133LM, and Scott A, respectively. The number of cells in the control sample remained approximately the same within 12 h of incubation.

### 3.7. Effects of Rhodomyrtus tomentosa Ethanolic Leaf Extract on Cells of Listeria monocytogenes Scott A, Determined by Scanning Electron Microscopy

**Figure 4:**
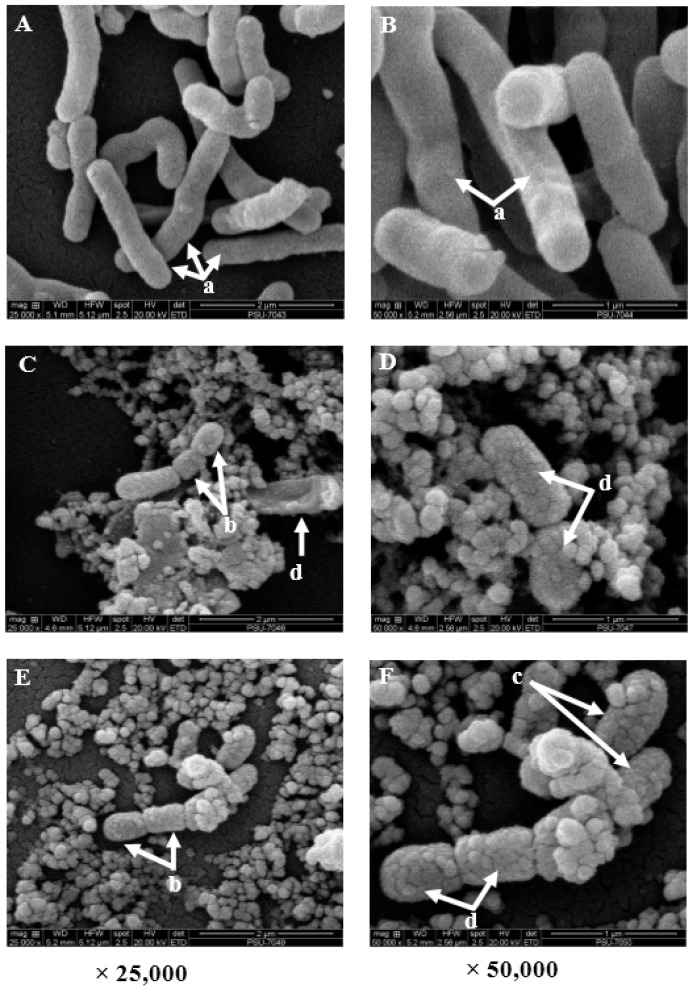
Scanning electron micrographs of *Listeria monocytogenes* Scott A cells exposed to *Rhodomyrtus tomentosa* ethanolic leaf extract for 12 h are depicted for the control (**A**,**B**); MIC (**C**,**D**); and 2 MIC (**E**,**F**). Normal long cells “a”, highly reduced cell sizes “b”, cells getting close to each other “c”, and cells displaying rough surfaces with degenerative changes “d”. Few to no cells were observed in the treated samples while the control sample demonstrated numerous cells. Magnification is 25,000× and 50,000×, respectively.

After 12 h of treatment with the plant extract at 32 µg/mL and 64 µg/mL, few to no cells were left in the treated samples (Figure 4). Treated cells displayed reduced sizes when compared with the longer cells observed in the control sample with the incorporation of 0.1% DMSO. Incomplete separation of cells or abnormal cell division was displayed by dying cells. Treated cells were found to come close to one another, which may suggest an attempt to increase cell survival. Surfaces of treated cells appeared rough and distorted with cracking degenerative changes, while the surfaces of cells in the control sample appeared smooth with no changes.

## 4. Discussion

Plant extracts are rich in aromatic substances and secondary metabolites, which have potencies as food preservatives. The present study was aimed at screening for a potential bio-control agent from *Rhodomyrtus tomentosa* against the foodborne pathogen *L. monocytogenes*. As observed in our study, antibiotic resistance in *L. monocytogenes* has been documented previously. Conter *et al.* [24] found resistance to 12 antibiotics in *L. monocytogenes* strains from food and food processing plant environments using the automated VITEK2 system. Our results revealed resistance to one or more commercial antibiotics as well as intermediate susceptibility to *L. monocytogenes* isolates from ready-to-eat food and food processing plant environments. Resistance genes in these isolates could be potentially transferable among strains, which may lead to increased therapeutic failure and constitute potential health hazards to consumers.

*R. tomentosa* ethanolic leaf extract displayed a highly remarkable antibacterial activity against *L. monocytogenes* isolates tested in the present study with low MIC_50_ and MIC_90_ values of 16 µg/mL and 32 µg/mL, respectively, when compared with results from previous studies. An earlier report found high MIC values from various plant leaf ethanol extracts against *L. monocytogenes* including mint timija (315 µg/mL), cinnamon (400 µg/mL), cistus (515 µg/mL), rose (900 µg/mL), thyme (1560 µg/mL), artemisia (3750 µg/mL), rosemary (5250 µg/mL), geranium (6150 µg/mL), chamomile (6750 µg/mL), lavender (11,500 µg/mL), and verberia (11,750 µg/mL) [10]. Furthermore, the ethanolic leaf extract of some plants belonging to the Myrtaceae family, including clove (250 µg/mL) [10], strawberry gum (500 µg/mL), lemon ironbark (500 µg/mL), ringwood (500 µg/mL), and lemon myrtle (>500 µg/mL) [25], showed higher MIC values against *L. monocytogenes* than *R. tomentosa* extract. The remarkable activity of *R. tomentosa* extract at lower concentrations has been previously reported against other food pathogenic bacteria including *Bacillus cereus* [14] and *Staphylococcus aureus* [15], with MICs ranging from 16 to 64 µg/mL and 32 to 128 µg/mL, respectively. Apart from the presence of betulinic acid, and ursolic and aliphitolic acids [26] in *R. tomentosa* leaves, several other compounds have been isolated from the leaves. These include triterpenoids [26], hydrolysable tannins [27], rhodomyrtone [28], and quercetin [29], all of which exhibit potent antibacterial properties. These bio-active components may be implicated in the antibacterial activity demonstrated by the extract in the present study.

The results obtained from the time-kill analysis showed that the extract demonstrated an overall bactericidal activity against *L. monocytogenes*. Some studies have documented the bactericidal activity of *R. tomentosa* extracts against other pathogens using the time-kill assay. Vegetative cells and endospores of *Bacillus cereus* were reduced by at least 3-log units within 6 to 8 h and 2 h, respectively, after incubation with *R. tomentosa* ethanolic leaf extract at 2 MIC and 4 MIC [14]. Also, a bioactive compound isolated from *R. tomentosa* leaf (rhodomyrtone) at 4 MIC reduced 99.9% of the initial cell numbers of *Staphylococcus aureus* in 3 h [15].

In addition, NaCl can constitute an osmotic stress condition to contaminating microbial cells in food and this is a commonly used hurdle in the food industry. However, *L. monocytogenes* is capable of surviving and proliferating in the presence of high salt concentrations, which has lead to long-term adaptation to sublethal and stress conditions. In the present study, tolerance of *L. monocytogenes* to 10% NaCl was highly reduced. A number of genes upregulated by the alternate sigma (σ) factor (σ^B^) are linked to the survival of *L. monocytogenes* in harsh environments. These genes were hypothesized to biosynthesize exopolisaccharides, which function in the modification of cell wall structure against high osmolarity [30]. Sublethal injury in microbial cells can distort the normal osmoregulating potential of the cell membrane and the ability of cells to exclude toxic substances. In our study, the reduction of salt tolerance in *L. monocytogenes* could be indicative of membrane damage in the cells, caused by the activity of the plant extract, thereby altering the cell membrane function as an osmotic barrier*.* A similar observation was reported by Carson *et al.* [20]. Treatments with tea tree oil and its compounds induced sublethal injury in the cell membrane of *Staphylococcus aureus*, causing loss of survival in growth medium incorporated with 5% and 7.5% (*w*/*v*) NaCl. The observations in our study may be a valuable insight into the effectiveness of multiple hurdle technology, using the plant extract in the presence of certain salt concentrations to control *L. monocytogenes* in food.

Microbial cells in the stationary phase of growth are generally less sensitive to injury from antibiotics than those in the exponential phase of growth [31]. In the present study, *R. tomentosa* ethanolic leaf extract demonstrated a remarkable activity against *L. monocytogenes* cells in the stationary phase of growth. Antimicrobial agents that inhibit the macromolecular synthetic process in bacteria, such as beta lactams and quinolones, often have minute effects on organisms in the stationary phase of growth. However, scanning electron micrographs of *L. monocytogenes* cells treated with *R. tomentosa* extract showed reduced cell sizes with abnormal cell division. This mechanism appears to be closely related to the mechanism of aminoglycosides’ action, majorly involving the inhibition of protein synthesis in microbial cells [32]. The activity of the extract on stationary phase *L. monocytogenes* cells may therefore be an effect of several bioactive components in the extract with different mechanisms of action. Micrographs of treated cells showed that exposure to plant extract had rigorous effects on the viability of *L. monocytogenes* cells. Treated cells were positioned close together, which may be a strategy to increase survival [33], and cell surfaces appeared rough and distorted with cracking degenerative changes which were absent in untreated cells. These observations correlated well with salt tolerance inhibition results. The disruption of cell membrane integrity may have resulted in the loss of osmoregulation potential [20], thereby causing cell degeneration [34]. A previous study reported abnormal binary fission and irregular shapes in *Streptococcus pyogenes* cells treated with *R. tomentosa* ethanolic leaf extract. However, treated cells maintained cell wall structures similar to untreated cells in the study [35]. Sianglum *et al.* [13] observed damage to cell wall- and membrane-related proteins in *Staphylococcus aureus* by the subinhibitory concentration of rhodomyrtone, a pure compound isolated from *R. tomentosa* leaves. Interestingly, the observations were comparable to results found in our study. Furthermore, essential oil from the leaves of *Macleaya cordata* altered cell wall and membrane integrity in *Ralstonia solanacearum* [34], with a cell appearance similar to that of treated *L. monocytogenes* observed in our study.

*R. tomentosa* leaves, roots, buds, and fruits have been used as traditional treatments for inflammatory and infectious conditions such as diarrhea, dysentery, colitis, abscesses, and hemorrhages in Vietnam, China and Malaysia [36], and Thailand and Bangladesh [37,38] over a long period of time. Also, the extract has no effect on Gram-negative organisms which represent the majority of the intestinal microflora. The extract may therefore pose no significant risk to indigenous gut flora. Earlier published data indicated that toxicity of *R. tomentosa* ethanolic leaf extract on skin cells is very low. The IC_50_ of the extract was 476 µg/mL, which is approximately 15 to 30 folds of the MIC values (16 to 32 µg/mL) in the present study [39]. Reports on the cytotoxicity of compounds extracted from *R. tomentosa* leaves, tested on human embryonic kidney cells, demonstrated no toxicity up to the highest dose [40]. Overall, *R. tomentosa* ethanolic leaf extract appears to interfere with cell growth and cell division as well as disrupt cell membrane integrity in *L. monocytogenes*. The extract shows promise as a natural alternative to synthetic food antimicrobials to prevent the incidence of *L. monocytogenes* contamination.

## 5. Conclusions 

The development of control measures for *L. monocytogenes* food contamination is important and still ongoing. Our study demonstrated the remarkable activity of *R. tomentosa* ethanolic leaf extract in inactivating this pathogen. The extract shows promise for further development into a bio-control agent against *L. monocytogenes*. *R. tomentosa* extract could be applied as a natural food additive to guard against hazards associated with *L. monocytogenes* food contamination without compromising food safety.
